# RhMED15a-like, a subunit of the Mediator complex, is involved in the drought stress response in *Rosa hybrida*

**DOI:** 10.1186/s12870-024-05059-8

**Published:** 2024-04-30

**Authors:** Nanxin Xie, Haoyang Shi, Xiaoman Shang, Zixin Zhao, Yan Fang, Huimin Wu, Ping Luo, Yongyi Cui, Wen Chen

**Affiliations:** Collaborative Innovation Center for Efficient and Green Production of Agriculture in Mountainous Areas of Zhejiang Province, Key Laboratory of Quality and Safety Control for Subtropical Fruit and Vegetable, Ministry of Agriculture and Rural Affairs, College of Horticulture Science, Zhejiang A&F University, Hangzhou, 311300 China

**Keywords:** *Rosa hybrida*, Drought tolerance, *RhMED15a-like*, Mediator, Phytohormone

## Abstract

**Background:**

Rose (*Rosa hybrida*) is a globally recognized ornamental plant whose growth and distribution are strongly limited by drought stress. The role of Mediator, a multiprotein complex crucial for RNA polymerase II-driven transcription, has been elucidated in drought stress responses in plants. However, its physiological function and regulatory mechanism in horticultural crop species remain elusive.

**Results:**

In this study, we identified a Tail module subunit of Mediator, *RhMED15a-like*, in rose. Drought stress, as well as treatment with methyl jasmonate (MeJA) and abscisic acid (ABA), significantly suppressed the transcript level of *RhMED15a-like*. Overexpressing *RhMED15a-like* markedly bolstered the osmotic stress tolerance of *Arabidopsis*, as evidenced by increased germination rate, root length, and fresh weight. In contrast, the silencing of *RhMED15a-like* through virus induced gene silencing in rose resulted in elevated malondialdehyde accumulation, exacerbated leaf wilting, reduced survival rate, and downregulated expression of drought-responsive genes during drought stress. Additionally, using RNA-seq, we identified 972 differentially expressed genes (DEGs) between tobacco rattle virus (TRV)-*RhMED15a-like* plants and TRV controls. Gene Ontology (GO) analysis revealed that some DEGs were predominantly associated with terms related to the oxidative stress response, such as ‘response to reactive oxygen species’ and ‘peroxisome’. Furthermore, Kyoto Encyclopedia of Genes and Genomes (KEGG) enrichment highlighted pathways related to ‘plant hormone signal transduction’, in which the majority of DEGs in the jasmonate (JA) and ABA signalling pathways were induced in TRV-*RhMED15a-like* plants.

**Conclusion:**

Our findings underscore the pivotal role of the Mediator subunit *RhMED15a-like* in the ability of rose to withstand drought stress, probably by controlling the transcript levels of drought-responsive genes and signalling pathway elements of stress-related hormones, providing a solid foundation for future research into the molecular mechanisms underlying drought tolerance in rose.

**Supplementary Information:**

The online version contains supplementary material available at 10.1186/s12870-024-05059-8.

## Background

Drought, one of the most common environmental stresses, has profound adverse effects on both plant growth and crop productivity, posing a grave threat to global sustainable agriculture [1–3]. Generally, moderate drought conditions impair biosynthetic capacity and nutrient acquisition, stunt growth, induce early flowering, and drastically diminish overall plant yield. Severe drought can result in irreparable damage or even plant death [1, 4, 5]. Plants have evolved myriad strategies to enhance survival during water deficit conditions, such as physiological and biochemical adaptations. Drought can stimulate the synthesis of stress-protective compounds such as trehalose and proline, activate antioxidant defence systems to maintain redox balance, and lead to the utilization of peroxidase enzymes to minimize cell injury and preserve membrane integrity [4, 6–8]. Moreover, drought stress induces modifications in the concentrations of various phytohormones, including abscisic acid (ABA) and jasmonate (JA), and their associated signalling networks. Many such adaptive responses involve systemic transcriptional regulation of genes critical to plant drought resistance [9, 10]. DNA-binding transcription factors (TFs) including dehydration-responsive element binding (DREB) protein, NAM ATAF CUC2 (NAC2), AP2/ERF, and WRKY, are well studied [9]. Nevertheless, the transcriptional regulation of downstream genes by gene-specific TFs calls for the participation of the Mediator complex as a coregulator, an indispensable facilitator in forwarding information from TFs to the transcription machinery [11, 12].

The Mediator complex acts as a molecular link between DNA-binding TFs and RNA polymerase II (RNA pol II) [13]. Initially discovered in yeast, Mediator has proven to be essential for in vitro transcription activation [14, 15]. Mediator proteins in human and plant cells were subsequently purified [16, 17]. Although earlier findings suggested low sequence conservation among Mediator subunits of diverse species, a later examination of genomic sequences from multiple eukaryotes indicated that Mediator is broadly evolutionarily conserved [18]. Eukaryotic Mediator consists of three core subcomplexes and a detachable kinase module [19, 20]. The core Mediator contributes to numerous transcriptional stages, including RNA pol II initiation, pausing and elongation, reinitiation, and transcription of certain siRNA and miRNA precursors [21, 22]. Among the core Mediator, the Tail subcomplex is considered to have a crucial function in its interaction with TFs [23–25].

Mediator subunits have been found to have functions in plant response pathways to environmental stimulus, such as cold, heat, salinity, drought, and nutrient deficiency [11, 19, 26–30]. MED25 has been shown to be a crucial element in stress response signalling pathways [11, 31, 32]. In *Arabidopsis*, while *med25* is sensitive to salt stress, it is resistant to drought [33–35]. The multifunctional protein MED25 interacts with various TFs. Genetic evidence revealed that MED25 acts as a positive modulator of JA signalling by interacting with MYC2 while also acting as a negative modulator of ABA signalling by interacting with ABI5 [32]. The ACID domain of MED25 also interacts with known drought-related TFs, such as DREB2A, MYB-like proteins, and ZFHD1 [34]. In *Arabidopsis*, *med18* exhibited heightened sensitivity to salt stress and ABA treatment. MED18 functions as a positive modulator of the ABA response and collaborates with ABI4 and YY1 to regulate the expression of important genes responsive to abiotic stresses [36–38]. The kinase module subunit CDK8 has been suggested to be an essential regulator of drought response pathways and ABA signalling in *Arabidopsis*. Compared with wild-type *Arabidopsis*, the *cdk8* mutant exhibited decreased ABA sensitivity, dysfunctional stomatal apertures, and heightened drought sensitivity [39]. Although extensive exploration of Mediator has been performed with *Arabidopsis*, studies involving horticultural crop species are limited.

Rose (*Rosa hybrida*) holds a significant place in the world due to its extensive use in ornamental gardens, as cut flowers, and as a crucial raw material for the perfume and cosmetic industries [40]. Despite its importance, cultivating roses is strongly hindered by drought stress, which restricts growth and blooming and considerably increases cultivation costs [41]. Unfortunately, the molecular mechanisms by which rose plants respond to drought stress are still largely ambiguous. Our previous research elucidated the function of the transcription factor RhPTM, which contributes to a striking balance between growth and drought survival in rose through interaction with the aquaporin RhPIP2;1 [42]. Here, we identified *RhMED15a-like*, a Tail module subunit of Mediator, which plays a crucial role in drought tolerance in rose. Furthermore, *RhMED15a-like* was found to control the expression of genes responsive to drought and those related to hormone signal transduction. The data from this study provide new details related to the involvement of the Mediator subunit in the plant response to abiotic stress and offer new insights into the molecular mechanics of drought stress tolerance in rose.

## Materials and methods

### Plant materials and growth conditions

The plantlets of *Rosa hybrida* cv. Samantha were obtained through tissue culture. The cultured shoots were nurtured on Murashige and Skoog medium (MS; Duchefa Biochemie) that included 1.0 mg/L 6-benzylaminopurine, 0.05 mg/L α-naphthaleneacetic acid, and 3 mg/L gibberellin A3 for a period of 30 days at a consistent temperature of 22 ± 1 °C, following a 16-h light and 8-h dark cycle. Subsequently, the shoots were moved to 1/2 MS medium that contained 0.1 mg/L α-naphthaleneacetic acid for 45 days to facilitate rooting. After successful rooting, the plants were moved to pots that contained peat moss and vermiculite (1:1) and grown under the same conditions as those described above for 30 days.

Transgenic *Arabidopsis thaliana* plants were derived from the Columbia (Col-0) ecotype background. These plants were cultivated in pots in an incubator under controlled conditions, i.e., 23 °C temperature, a photoperiod of 16/8 h (light/dark), and approximately 60% relative humidity.

### Dehydration and mannitol treatments

For the rose dehydration treatments, the cleaned roots of the plants were held in purified water for 1 day. Then, the plants were desiccated on the middle layer of a climate chamber set at a temperature of 25 °C, approximately 60% relative humidity, and a light intensity of 250 μmol·m^−2^·s^−1^. The control plants were maintained in water under the same conditions. Each plant was weighed and sampled at the specific time points during dehydration.

For mannitol stress treatment in transgenic *Arabidopsis*, approximately 36 seeds from each of the *RhMED15a-like*-overexpressing plant lines (OE-6, 8, and 9) and the vector control (VC) were cultivated on MS plates with varying mannitol concentrations (0, 200, and 300 mM). The germination rates were measured on the 9th day post-sowing. For the root length and fresh weight assays, sterilized seeds from both the OE and VC lines were nurtured on MS medium for three days. These seedlings were then moved to fresh medium supplemented with mannitol (0, 200, or 300 mM). Data regarding root length and plant fresh weight were collected after 12 days of vertical growth. Each treatment had three independent biological replicates.

### Phytohormone treatments

Rose leaf discs were soaked in 200 μM ABA, 200 μM methyl jasmonate (MeJA) or 200 μM salicylic acid (SA) solution plus 0.05% Tween for 1 d. Mock samples were treated with corresponding solution without any phytohormones. Then, these processed leaf discs were sampled and subjected to the next step of gene expression testing.

### RNA extraction and quantitative real-time PCR

An RNA rapid extraction kit (BOLAZ, Nanjing, China) was used to isolate total RNA from the rose leaves or roots. First-strand complementary DNA (cDNA) was generated by employing the PrimeScript™ II 1st Strand cDNA Synthesis Kit (Takara, Shiga, Japan). qRT‒PCR was performed by using the TaKaRa™ SYBR® FAST qPCR Kit (Takara, Shiga, Japan). The transcript levels were analysed with reference to *RhUBI2.* All reactions were biologically replicated a minimum of three times. Table S1 lists the primers utilized in this study.

### Vector construction and *Arabidopsis* transformation

The coding sequence of *RhMED15a-like* was incorporated into the KpnI and SalI sites of the pCAMBIA2300s vector. The resulting pCAMBIA2300s-*RhMED15a-like* plasmid was subsequently introduced into *Agrobacterium tumefaciens* strain GV3101. The recombinant plasmid was further transferred into wild-type (WT) *Arabidopsis* via the floral dipping method [43]. Transgenic *Arabidopsis* plants were screened on MS medium containing 50 mg/L kanamycin. Subsequently, transgenic lines that showed 100% germination were chosen for further use. The expression levels of *RhMED15a-like* in each selected line of the T_3_ generation were evaluated by qRT‒PCR. Table S1 lists the primers utilized in this study.

### Subcellular localization

The ORF sequence of *RhMED15a-like* was integrated with the carboxyl-terminal side of the GFP gene before being inserted into the pCAMBIA2300s vector. The use of a plasma membrane marker (PM-rk) and nuclear marker (NF-YA4) labelled with mCherry has been reported. Subsequently, both pCAMBIA2300s-*GFP-RhMED15a-like* and the two abovementioned marker vectors were transformed separately into *Agrobacterium* strain GV3101. The leaves of tobacco plants (*Nicotiana benthamiana*) that were 5 weeks old were infiltrated with *Agrobacterium*. A laser scanning confocal microscope (Olympus, Tokyo, Japan) was used to detect the fluorescence signals.

### Virus-induced gene silencing (VIGS)

VIGS of *RhMED15a-like* was implemented in accordance with a previously described method [44]. A 350-base pair (bp) specific fragment from the ORF region of *RhMED15a-like* was inserted into the pTRV2 vector. The pTRV2-*RhMED15a-like* vector, along with the pTRV1 and pTRV2 vectors, was introduced into *Agrobacterium* strain GV3101. After incubating, cells of *Agrobacterium* were collected and resuspended in infiltration buffer to an OD_600_ of approximately 1.5. Rose plantlets were immersed in infiltration buffer and subjected to a vacuum pressure of -25 kPa twice, each lasting for 60 s. Next, the soaked plants were washed and then transplanted into pots for further examination.

Regarding the drought stress treatment, *Agrobacterium-*infected plants were cultivated at a normal growth conditions for 30 days. Then, the plants were deprived of water for 20 days and allowed to return to regular watering for another 20 days. Observations of any phenotypical variations in the rose plants were made at several time intervals.

### Transcriptomic sequencing and analysis

Leaves of TRV control and TRV-*RhMED15a-like* plants subjected to RNA sequencing (RNA-seq) were harvested 45 days after infection Leveraging Sequencing by Synthesis (SBS) technology, a significant amount of high-quality raw data was acquired utilizing an Illumina NovaSeq 6000 to produce 150 bp paired-end reads. After data processing, the raw sequences were converted into clean reads, which were subsequently aligned to the reference genome sequence (*Rosa chinensis* GCA_002994745.1_RchiOBHm_V2 from NCBI). The relative expression levels were calculated using FPKM, which represents the anticipated count of fragments per kilobase of transcript sequence per million mapped fragments. The derived *p* values were obtained using the Benjamini and Hochberg method to control the false discovery rate. The correlation coefficient and principal component analysis between every pair of biological replicates were calculated (Fig. S3). Genes that exhibited a |log2FC|≥ 1.0 and a *p* value ≤ 0.05 were regarded as significantly differentially expressed genes (DEGs). Furthermore, Cluster Profiler was used for enrichment analysis to statistically categorize the DEGs in the Gene Ontology (GO) and Kyoto Encyclopedia of Genes and Genomes (KEGG) pathways.

### Statistical analysis

Significant discrepancies between the experimental data related to the physiological indices and the results from the qRT‒PCR analysis were assessed using either one-way analyses of variance (ANOVAs) with Duncan’s multiple range tests or independent-samples t test (*p* < 0.05).

## Results

### Characteristics of *RhMED15a-like*

**Fig. 1:**
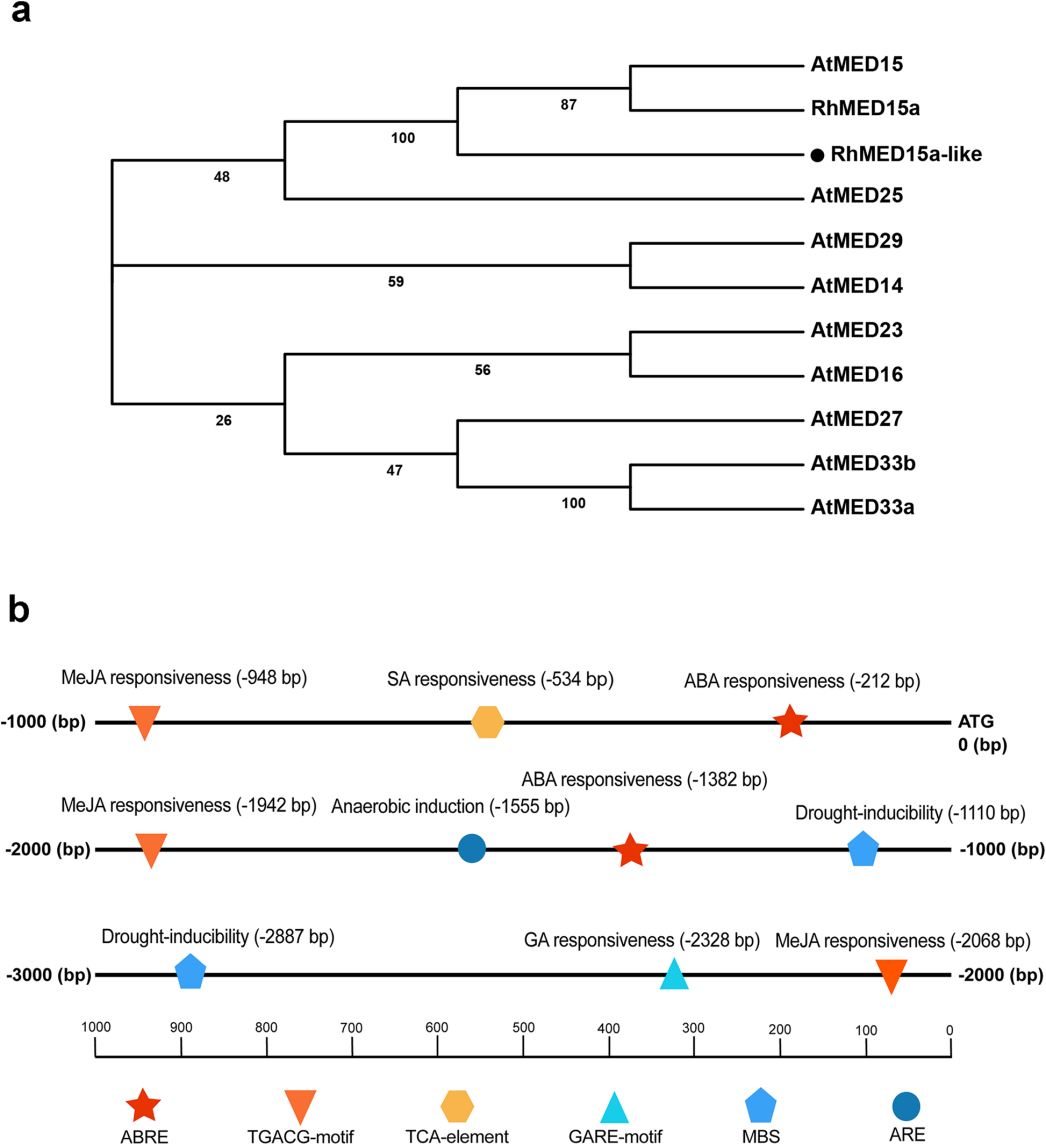
Phylogenetic relationships of RhMED15a-like and diagram of the *RhMED15a-like* promoter region. **a** Phylogenetic relationships between RhMED15a-like, RhMED15a and the Mediator Tail module subunits in *Arabidopsis*. MEGA 7.0 was utilized to construct the neighbour-joining tree. The RhMED15a-like protein is represented by a black solid circle. Bootstrap values delineate each branch's divergence, with the scale demarcating the branch length. AtMED15 (NP_173030.1), AtMED25 (NP_173925.3), AtMED29 (NP_172641.2), AtMED14 (NP_187125.1), AtMED23 (NP_173737.1), AtMED16 (NP_192401.5), AtMED27 (NP_566345.1), AtMED33b (NP_566125.4), AtMED33a (NP_189001.1). **b** Potential CREs present in the *RhMED15a-like* promoter. The CREs that respond to plant hormones and environmental stresses were analysed using the PlantCARE database and are indicated by different colours

In our previous work, we discovered that the transcript level of a gene encoding a Mediator subunit, RhMED15a-like, in rose petals was significantly reduced under dehydration stress, as indicated by the transcriptome data (Fig. S1). Subsequently, we successfully isolated the full-length cDNA sequence of *RhMED15a-like* from rose. The ORF of *RhMED15a-like* spans 1590 base pairs and encodes a protein consisting of 529 amino acids. To investigate the structural features of RhMED15a-like, we analysed its sequence using the Interpro database and TMHMM web servers [45, 46]. The results revealed the presence of a KIX domain and a transmembrane domain within the deduced protein sequence of RhMED15a-like (Fig. S2). Additionally, upon aligning the deduced amino acid sequences of RhMED15a-like with those of its homologues in other plants, we observed a high degree of similarity at the amino terminus, which contains the conserved KIX domain. This domain is known to be involved in interactions between Med15 and various transcriptional activators in *Arabidopsis* [47]. Notably, RhMED15a-like was the only protein that featured a transmembrane domain at the carboxyl terminus (Fig. S2). Through phylogenetic analysis between RhMED15a-like, RhMED15a (another homologous protein of MED15 in rose) and the Mediator Tail module subunits in *Arabidopsis thaliana*, including MED14, MED15, MED16, MED23, MED24/33/5, MED25, MED27/3, and MED29/32/2 [22], we found a close relationship between RhMED15a-like, RhMED15a and AtMED15 (Fig. 1a). These findings suggest that RhMED15a-like is a homologue of the MED15 protein and belongs to the Mediator Tail module, which plays a crucial role in interacting with gene-specific TFs (Fig. 1a) [47].

Then, we obtained a nearly 3 kb promoter sequence of *RhMED15a-like* and conducted an analysis of *cis*-acting regulatory elements (CREs) using the PlantCARE database. The results indicated the presence of several CREs in the *RhMED15a-like* promoter that respond to plant hormones and abiotic stresses (Fig. 1b). These included two ABA-responsive elements (ABREs), three MeJA-responsive elements (TGACG motifs), one SA-responsive element (TCA element), and one gibberellin (GA)-responsive element (GARE motif) for phytohormone response. Additionally, there were two drought-responsive MYB binding sites (MBSs) and one anaerobic stress-responsive element (ARE) involved in the abiotic stress response. These results suggested that the transcriptional profile of *RhMED15a-like* may vary under drought stress conditions and in the presence of certain hormones, such as ABA, MeJA, SA, and GA.

### Analyses of the expression of *RhMED15a-like* in response to drought and phytohormone treatments

**Fig. 2:**
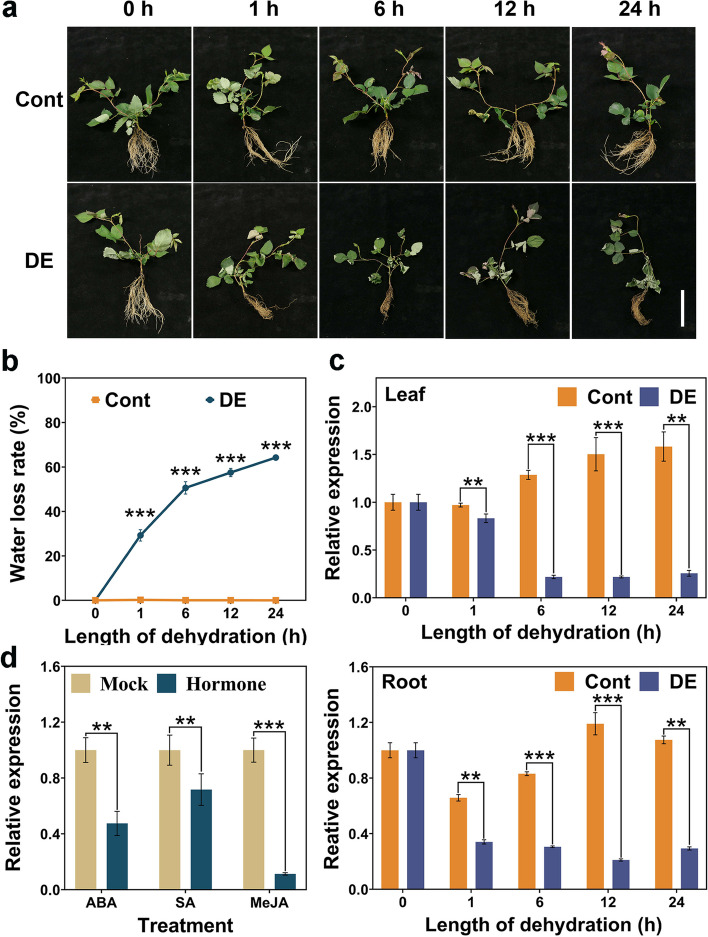
The expression level of *RhMED15a-like* in rose plants under dehydration stress and hormone treatments. **a** Morphological changes in rose plants under dehydration treatment. Dehydration-treated rose plants were placed horizontally with their roots exposed to air, while the controls were held in water under the same conditions. Cont, control plants; DE, dehydration-treated plants. Scale bar, 7 cm. **b** The water loss rate of rose plants under dehydration stress. The values represent the means ± SDs (*n* = 3). **c** Relative expression of the *RhMED15a-like* gene in the leaves and roots of rose plants after dehydration treatment for 0, 1, 6, 12, and 24 h. The transcript level of *RhMED15a-like* at 0 h was normalized to 1. The values represent the means ± SDs (*n* = 3). **d** Relative expression of *RhMED15a-like* under exogenous hormone treatments. ABA, 200 µM abscisic acid; SA, 200 µM salicylic acid; JA, 200 µM methyl jasmonate. The values represent the means ± SDs (*n* = 3). The asterisks denote significant differences calculated using the independent samples t test (**p < 0.01; ***p < 0.001, two-tailed)

To further analyse the responsiveness of *RhMED15a-like* to water stress, we examined the expression profile of *RhMED15a-like* in 1-month-old rose seedlings subjected to 24 h of dehydration. As depicted in Fig. 2a, the control plants displayed healthy characteristics, with smooth leaves and light brown roots. In contrast, plants exposed to dehydration stress exhibited symptoms of wilting and drying after just 1 h of treatment. Furthermore, some leaves became curved, and the roots darkened in colour. Consistent with these observations, the dehydration-treated plants experienced a significantly greater rate of water loss than did the control plants during the treatment period (Fig. 2b). The qRT‒PCR results indicated that *RhMED15a-like* transcription was rapidly and significantly repressed by dehydration stress in both the leaf and root samples of rose plants (Fig. 2c). The abundance of *RhMED15a-like* decreased substantially in both organs within 1 h of treatment, reaching approximately 14.5% in leaves and 17.7% in roots after 12 h compared to that in the control. These findings aligned with the transcriptome data from dehydrated rose petals (Fig. S1), suggesting that the inhibitory effect of dehydration treatment on *RhMED15a-like* expression extends to multiple organs in rose.

We also determined the transcript level of *RhMED15a-like* in response to drought-related phytohormones, including ABA, MeJA, and SA [48–50], based on the analysis of CREs of its promoter (Fig. 1b). Leaf discs were soaked in solutions of these phytohormones for 24 h. The results demonstrated a significant downregulation of *RhMED15a-like* by ABA, MeJA, and SA after this treatment period (Fig. 2d). Specifically, MeJA and ABA treatments reduced *RhMED15a-like* expression by approximately 88.7% and 52.6%, respectively, suggesting a potential role for *RhMED15a-like* in MeJA- and ABA-mediated stress responses.

### RhMED15a-like was localized to the nucleus and plasma membrane

**Fig. 3:**
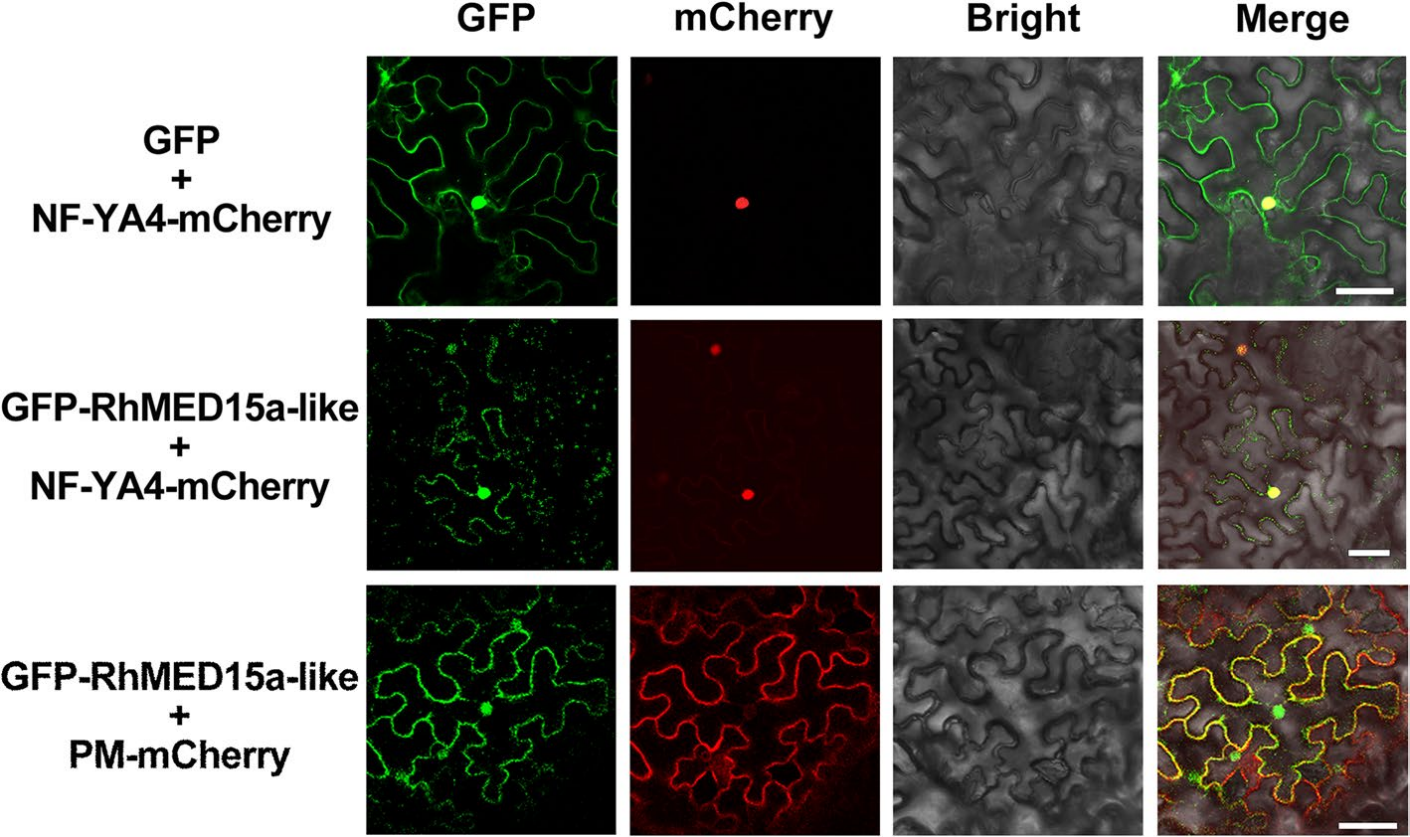
Subcellular localization of RhMED15a-like.GFP-RhMED15a-like signals expressed in *N. benthamiana.* It was expressed alongside both the plasma membrane marker (CD3-1007) and the nuclear marker (NF-YA4), each labelled with mCherry. GFP paired with NF-YA4-mCherry served as the control. Scale bars, 50 μm

To trace the subcellular localization of RhMED15a-like, we obtained a 35S::*GFP-RhMED15a-like* construct and introduced it into the leaf cells of *Nicotiana benthamiana*. mCherry-labelled plasma membrane markers or nuclear markers were also used [51]. The results showed that the green fluorescence signals of RhMED15a-like closely matched the red signals of both markers (Fig. 3), indicating that RhMED15a-like was localized to the nucleus and plasma membrane.

### Overexpression of *RhMED15a-like* enhanced osmotic stress tolerance

**Fig. 4:**
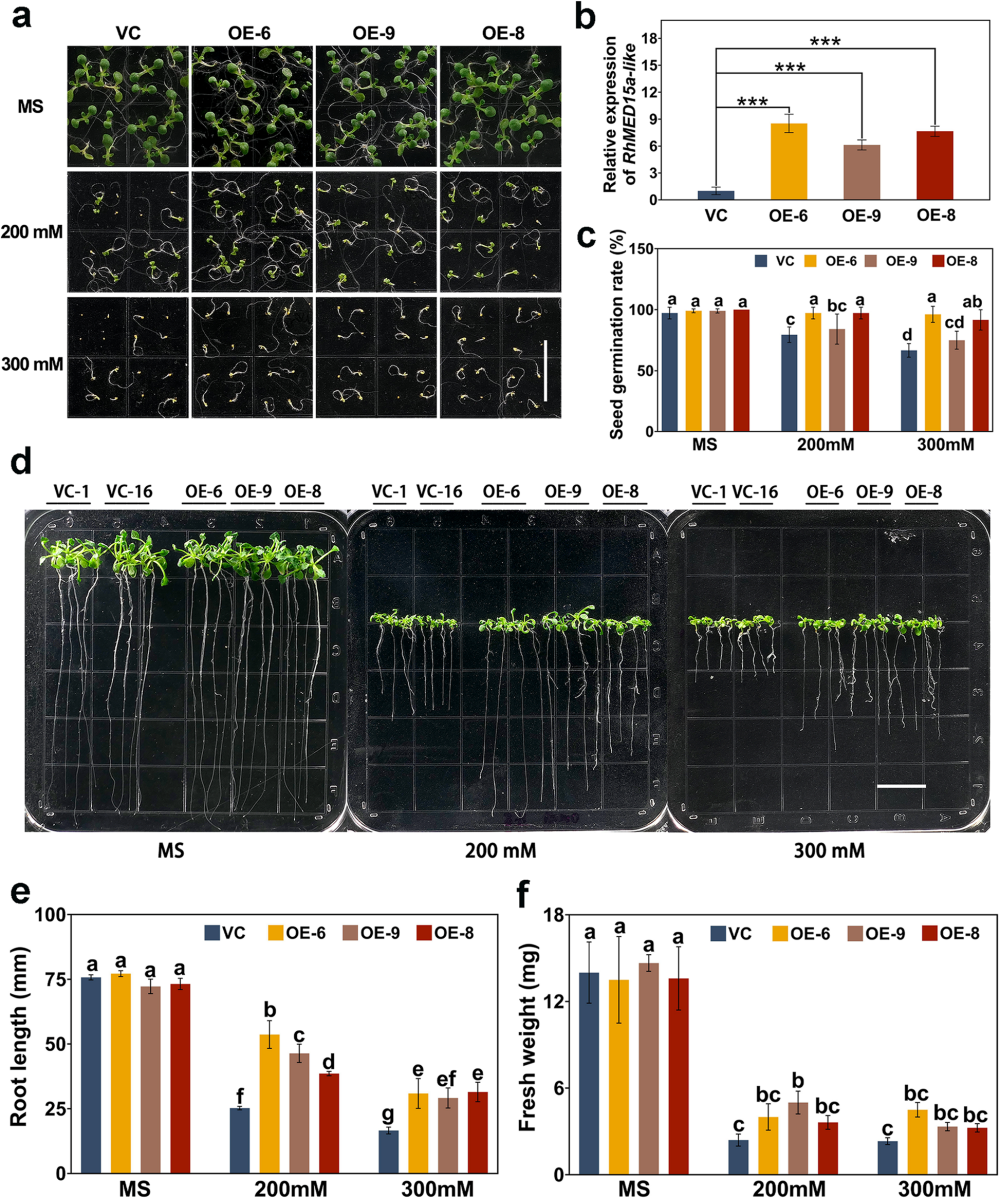
*RhMED15a-like* overexpression augmented osmotic stress tolerance in *Arabidopsis*. **a** Seed germination phenotype of *RhMED15a-like*-overexpressing (OE) lines and vector control (VC) plants. T_3_ seeds from *RhMED15a-like*-OE lines (OE-6, 8, and 9) and VC plants were subsequently grown on MS plates supplemented with mannitol (0, 200, or 300 mM). Images of the seedlings were taken nine days after they were planted. Scale bar, 1.5 cm. **b** Expression of *RhMED15a-like* in the leaves of 3-week-old *RhMED15a-like*-OE and VC plants.The values represent the means ± SDs (*n* = 3). Significant differences determined by independent samples t test are denoted by asterisks (** *p* < 0.01; *** *p* < 0.001, two-tailed). **c** Seed germination rates of *RhMED15a-like*-OE and VC plants subjected to osmotic stress. The results are based on three replicates with 36 seeds per line. **d** Root phenotype of the *RhMED15a-like*-OE and VC lines after osmotic stress treatment. Both groups were nurtured for 3 days and then transferred to MS agar plates containing varying mannitol concentrations (0, 200, and 300 mM). Scale bar, 1.5 cm. Root length (**e**) and fresh weight analysis (**f**) of *RhMED15a-like*-OE and VC plants. The root length and fresh weight were determined after 12 days of vertical growth. Statistically significant differences were analysed using Duncan’s multiple range test and are denoted with lowercase letters (*p* < 0.05)

To test the possible involvement of *RhMED15a-like* in the drought tolerance of plants, we overexpressed *RhMED15a-like* in *Arabidopsis* via a constitutive promoter. Three stable and homozygous overexpression (OE) lines (OE-6, 8, and 9) with relatively high expression levels of *RhMED15a-like* were used for phenotypic analyses (Fig. 4b). For the osmotic stress treatment, seeds from both the OE and vector plant (VC) groups were placed on MS plates containing 0, 200 or 300 mM mannitol. On MS medium without mannitol, nearly 100% of the seeds in both groups germinated (Fig. 4a, c). However, the seed germination rates of both groups greatly decreased under osmotic stress. After the plants were exposed to 200 mM mannitol, the average germination rate of the OE plants was approximately 1.2-fold greater than that of the VC plants.

The phenotypes of the roots of *RhMED15a-like* OE plants under mannitol treatment were also analysed. Three-day-old plants were moved to MS medium containing varying concentrations (0, 200, and 300 mM) of mannitol, followed by a 12-day vertical growth period. In the absence of mannitol, the length of the primary roots of the OE plants were equal to those of the VC plants, as determined via observation on MS plates (Fig. 4d, e). An increase in the concentration of mannitol correspondingly decreased primary root lengths in all plants, with the greatest suppression observed at 300 mM mannitol. However, under osmotic stress, the OE plants exhibited significantly longer primary root lengths than did the VC plants (Fig. 4d, e). In the medium containing 200 mM mannitol, the root lengths of the three OE lines decreased by approximately 37.9%, in contrast to the average 66.7% reduction observed in the VC plants. In addition, compared with that of the VC plants, the fresh weight of the OE plants was significantly greater after mannitol exposure (Fig. 4f). Collectively, these results suggested that *RhMED15a-like* overexpression bolstered osmotic tolerance in *Arabidopsis*.

### Silencing of *RhMED15a-like* reduced tolerance to drought stress

**Fig. 5:**
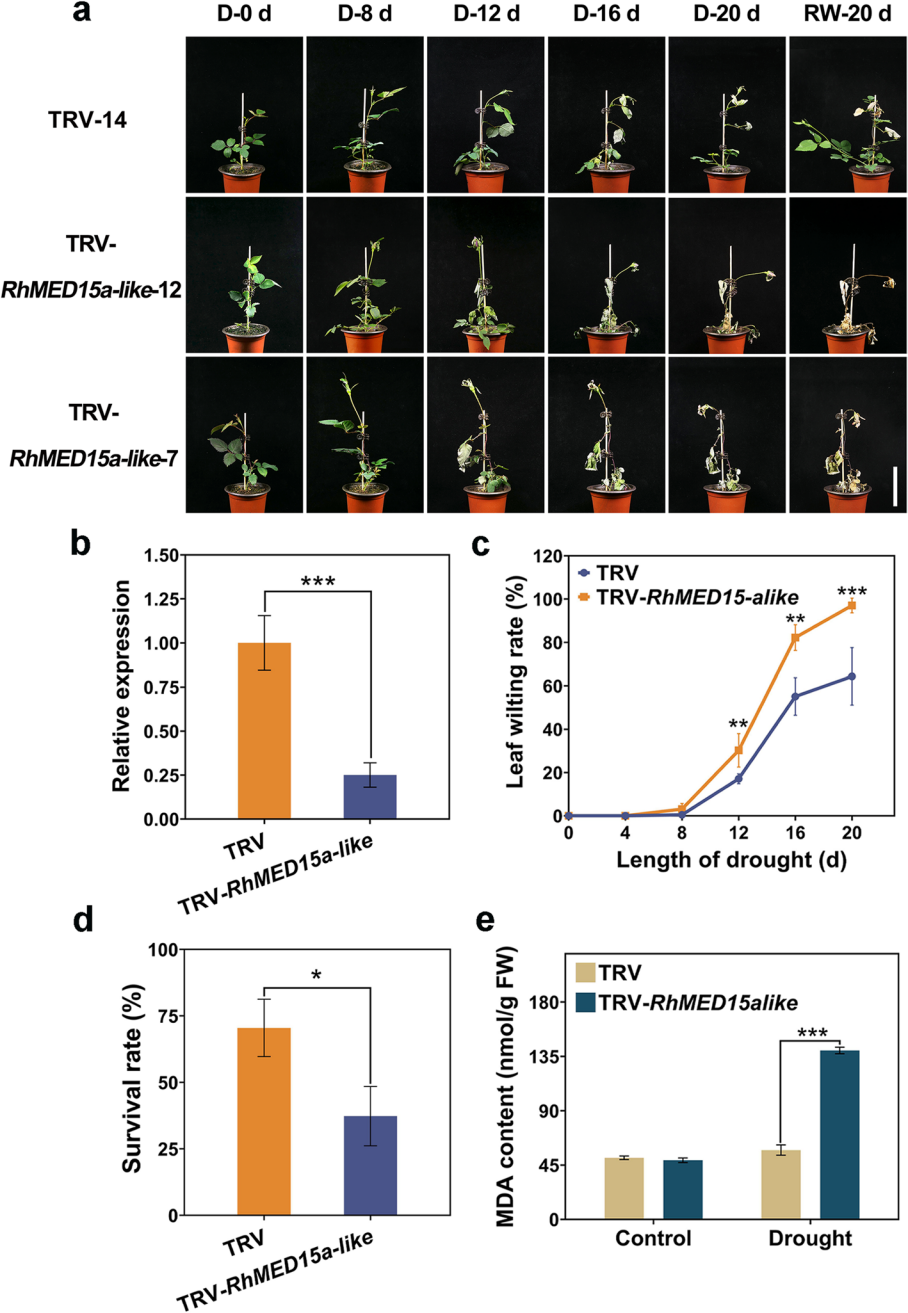
*RhMED15a-like* played a positive role in the resistance of roses to drought stress. Both TRV (TRV control) and TRV-*RhMED15a-like* plants underwent similar growth conditions: a 30-day period under normal conditions, a subsequent 20-day period of water deprivation, and a subsequent 20-day period of rewatering. **a** Phenotypes of TRV and TRV-*RhMED15a-like* during drought treatment. D, drought treatment; RW, rewatering stage. Scale bar, 7 cm. **b** Relative expression of *RhMED15a-like* in TRV and TRV-*RhMED15a-like* plants. *RhUBI2* served as the internal reference gene. Data are the means ± SDs (*n* ≥ 5). **c** Leaf wilting rate of TRV-*RhMED15a-like* under drought condition. Data are the means ± SDs (*n* ≥ 5). **d** The survival rate of TRV-*RhMED15a-like* after drought treatment. Data are the means ± SDs of 3 biological replicates. **e** MDA content of TRV-*RhMED15a-like* under drought conditions. Data are the means ± SDs (*n* ≥ 3). Significance of differences determined by independent samples t test are denoted by asterisks (*p < 0.05; **p < 0.01; ***p < 0.001, two-tailed)

To gain further insight into the role of *RhMED15a-like* under water scarcity, *RhMED15a-like* was silenced in rose plants using a VIGS approach. As demonstrated by the qRT‒PCR results, *RhMED15a-like* expression was substantially lower in the TRV-*RhMED15a-like* plants than in the TRV plants (Fig. 5b). For drought stress testing, both groups underwent 20 days of water deprivation on the 30th day post infection, and subsequent normal watering was resumed for another 20 days. Leaf wilting rates were documented every four days. Figure 5a shows that on Day 16 of drought treatment, the TRV control plants began to wilt. In comparison, the *RhMED15a-like* silenced plants exhibited wilting much earlier, on Day 12, and exhibited greatly increased wilting during the drought treatment. The leaf wilting rate of the *RhMED15a-like*-silenced plants increased strikingly under drought stress, reaching nearly 1.5 times that of the TRV control plants on Day 20 (Fig. 5c).

The MDA contents of both the control plants and those with *RhMED15a-like* silenced were compared before drought stress and after twelve days of treatment. Significant MDA variations were not detected between the two groups under normal conditions (Fig. 5e). However, the MDA content of the *RhMED15a-like*-silenced plants was approximately 2.4 times greater than that of the TRV plants during drought treatment, showing that these plants experienced more damage during drought treatment. Moreover, nearly 70.5% of the TRV control plants survived after rewatering, but only approximately 37.3% of the *RhMED15a-like*-silenced plants survived drought (Fig. 5d). These findings suggested that the silencing of *RhMED15a-like* decreases tolerance to drought stress in rose.

**Fig. 6:**
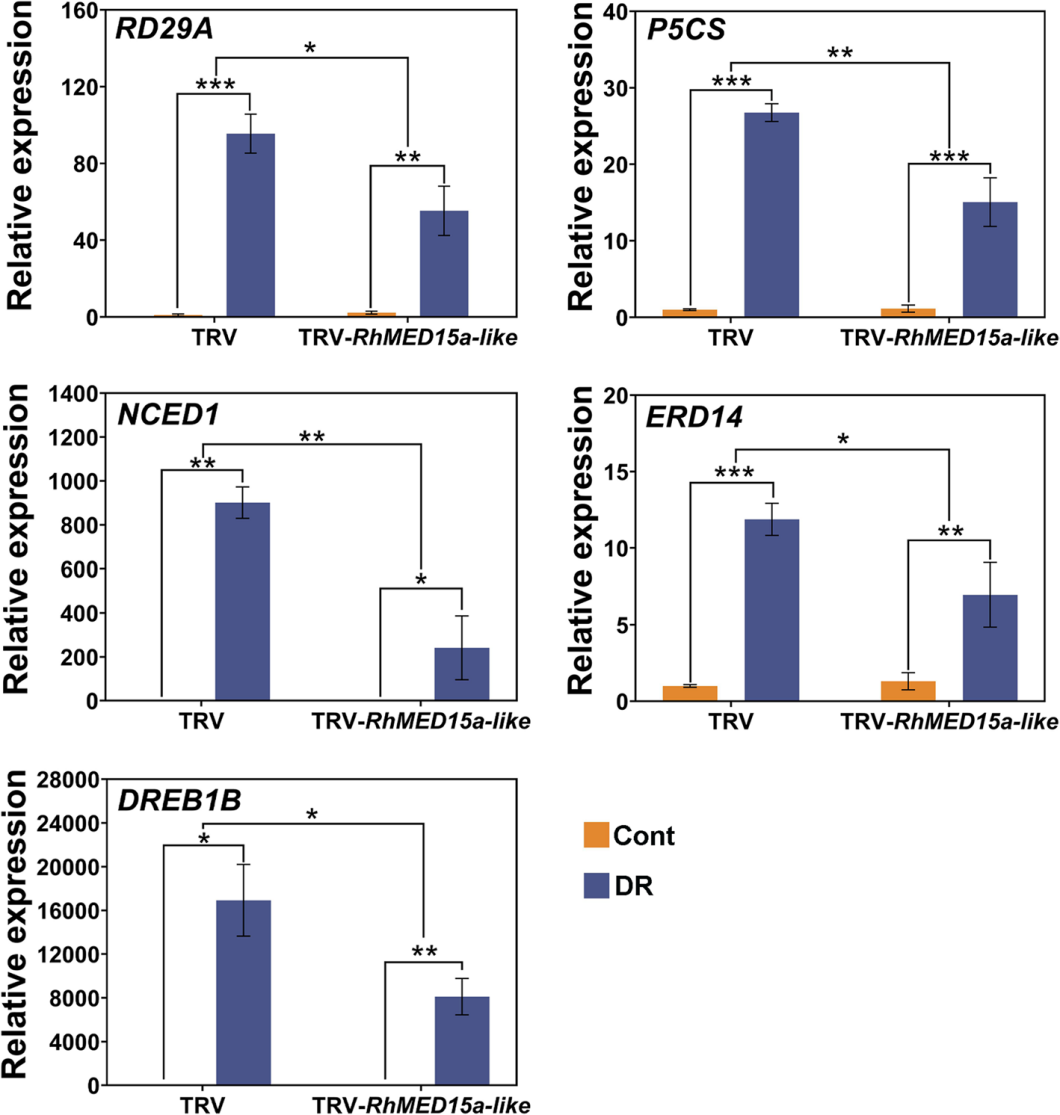
Silencing of *RhMED15a-like* downregulated expression of drought-responsive genes during drought stress. Quantitative RT‒PCR analysis of drought stress-responsive genes from the leaves of control plants and those with *RhMED15a-like* silenced before drought treatment (Cont) and after 20 days of treatment (DR). Data are the means ± SDs (*n* = 3). Significance of differences determined by independent samples t test are denoted by asterisks (* *p* < 0.05; ** *p* < 0.01; *** *p* < 0.001, two-tailed)

To explore the potential role of RhMED15a-like in enhancing the resistance of roses to water stress through the regulation of drought-related genes, we assessed the transcript levels of several drought-responsive genes, namely, *RD29A*, *P5CS*, *ERD14*, *NCED1*, and *DREB1B*, in control plants and those with *RhMED15a-like* silenced on the twentieth day of water deficit treatment. The results showed that the expression of the tested genes were substantially increased in both groups under drought condition. However, the drought-induced expression levels of these genes were significantly lower in the TRV-*RhMED15a-like* plants than in the TRV controls (Fig. 6), indicating that *RhMED15a-like* promotes the transcription of these genes during drought stress.

### Analysis of the regulatory network downstream of *RhMED15a-like*

**Fig. 7:**
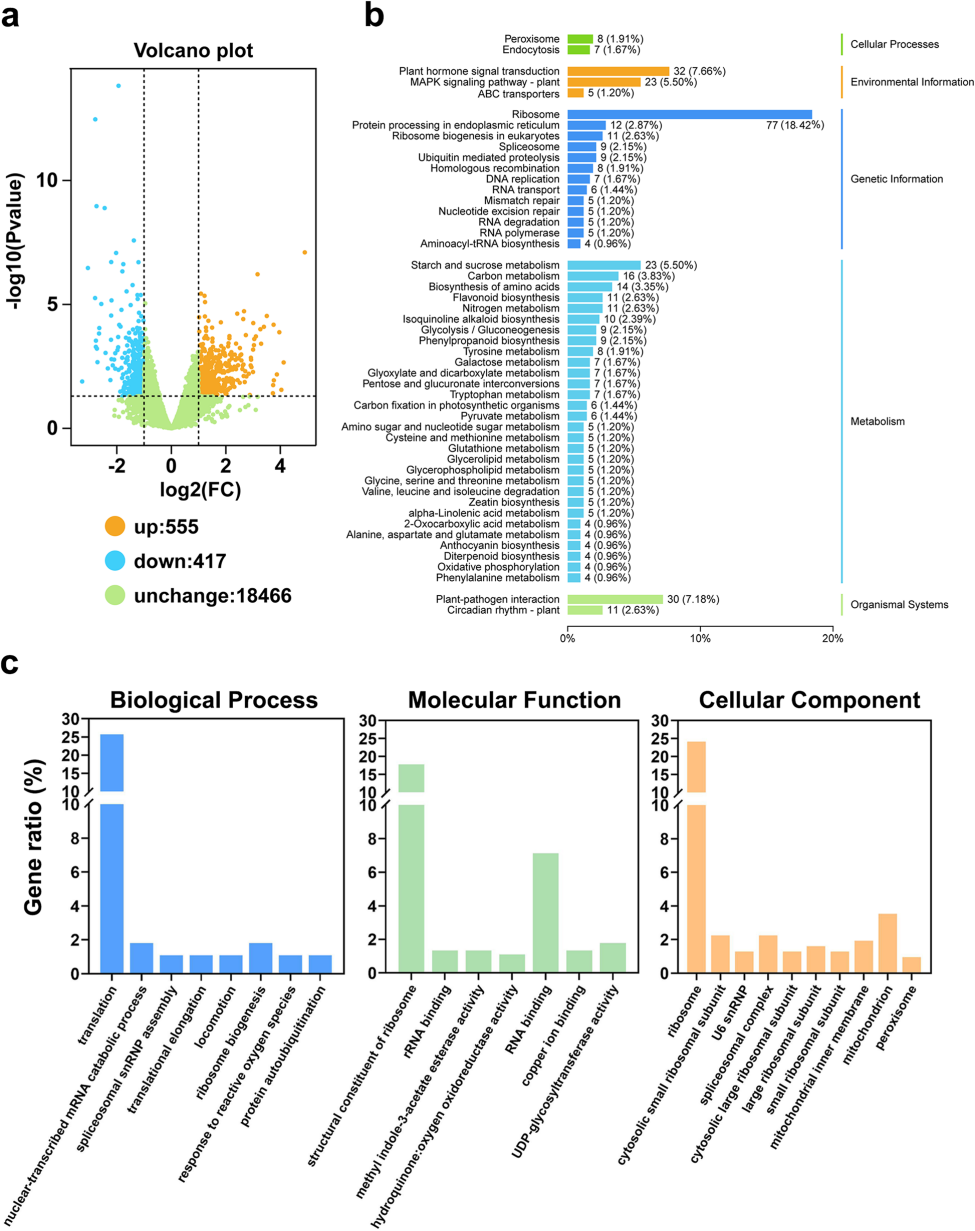
KEGG and GO analyses of DEGs between *RhMED15a-like*-silenced plants and TRV controls. **a** Volcano diagram analysis of the DEGs. A total of 972 DEGs were identified in the TRV-*RhMED15a-like* and TRV control comparisons, with 555 upregulated and 417 downregulated DEGs. **b** KEGG assignments for the DEGs. The number next to the column indicates the total number of DEGs in the same category, and the percentage in the bracket indicates the proportion of a specific category of DEGs to the total number of annotated DEGs. **c** GO assignments for the DEGs (*p* < 0.05, gene ratio > 1.0). The percentage of a specific category of DEGs in each main category is indicated by the left y-axis

To gain further insight into the regulatory network downstream of *RhMED15a-like,* we employed a large-scale screen for DEGs between TRV-*RhMED15a-like* and TRV plants via RNA-seq. After filtration, we obtained 37.7 Gb of clean data, averaging 6.3 Gb per sample. Clean reads were subsequently aligned to the reference genome of *Rosa chinensis* ‘Old Blush’. The Q30 levels of all the samples exceeded 92.0%, with the GC content of the six samples ranging between 47.4% and 48.2% (Table S2). We identified 972 DEGs (FDR ≤ 0.05 and |log2FC|≥ 1) between TRV-*RhMED15a-like* and TRV plants, among which 555 genes were upregulated and 417 genes were downregulated in TRV-*RhMED15a-like* plants compared to TRV plants (Fig. 7a).

The DEGs were mapped to the GO database to identify related functions (Fig. 7c). We observed significant enrichment of GO terms in the biological process (BP) category such as ‘translation’, ‘nuclear-transcribed mRNA catabolic process’, and ‘translational elongation’; terms in the molecular function (MF) category such as ‘structural constituent of ribosome’, and ‘RNA binding’; and terms in the cellular component (CC) category such as ‘ribosome’ and ‘spliceosomal complex’, revealing the potential roles of *RhMED15a-like* related to these GO terms, which was consistent with the functions of MEDs in numerous stages of transcription [21, 22]. We also observed significant enrichment of the GO terms ‘response to reactive oxygen species’ in the BP category, ‘hydroquinone:oxygen oxidoreductase activity’ in the MF category, and ‘peroxisome’ in the CC category, suggesting the possible involvement of *RhMED15a-like* in the response to oxidative stress, which can be triggered by drought conditions as a secondary stress [2]. The expression levels of most DEGs associated with these three GO terms were upregulated in the *RhMED15a-like*-silenced plants compared with those in the TRV control plants (Table S3). This result suggested that disruption of the *RhMED15a-like* gene may regulate oxidative stability in rose plants.

The analysis of the DEGs by employing KEGG pathway enrichment, facilitated by the use of the KOBAS program, indicated the enrichment of DEGs within several pathways. These pathways included ‘ribosome’, ‘plant hormone signal transduction’, ‘MAPK signalling pathway’, and ‘starch and sucrose metabolism’ (Fig. 7b). Phytohormones play an essential role in the response to abiotic stresses in plants, prompting us to pay particular attention to the thirteen DEGs that were involved in the signalling pathway of stress-associated hormones. These hormones included ABA, JA, and SA (Table 1). The ABA signal transduction pathways contained the greatest number of DEGs, followed closely by the JA signal transduction pathways. Notably, we identified the upregulation of most DEGs within the ABA and JA signalling pathways due to the silencing of *RhMED15a-like* in contrast to that in the TRV control. This result suggested that disruption of the *RhMED15a-like* gene influences the signal transduction of these stress-associated hormones in rose plants. This interference might be a leading factor altering rose drought resistance.

**Table 1:** DEGs involved in the signal transduction pathways of stress-related phytohormones

Gene ID	Gene description	log_2_FC	*p* value
**ABA**			
LOC112177157	serine/threonine-protein kinase SAPK2-like	-2.0995	0.0017
LOC112170995	transcription factor PIF1	1.1555	0.0076
LOC112185404	protein phosphatase 2C 77-like	2.0422	0.0030
LOC112186701	serine/threonine-protein kinase SAPK1-like	1.1803	0.0192
LOC112197995	major allergen Pruar 1-like	1.5827	0.0361
LOC112177387	low-temperature-induced 65 kDa protein isoform X1	2.0984	0.0004
**JA**			
LOC112189737	protein TIFY 10a	1.3313	0.0172
LOC112190005	protein TIFY 10b-like	2.7137	0.0050
LOC112176325	protein TIFY 6B isoform X1	1.1956	0.0161
LOC112175688	transcription factor MYC2-like	1.6827	0.0050
LOC112182139	transcription factor MYC3	1.1961	0.0017
**SA**			
LOC112170085	pathogenesis-related protein 1-like	-1.18125	0.0178
LOC112200867	pathogenesis-related protein PR-1-like	-1.08938	0.0430

TRV vs. TRV-*RhMED15a-like*

*FC* fold change

**Fig. 8:**
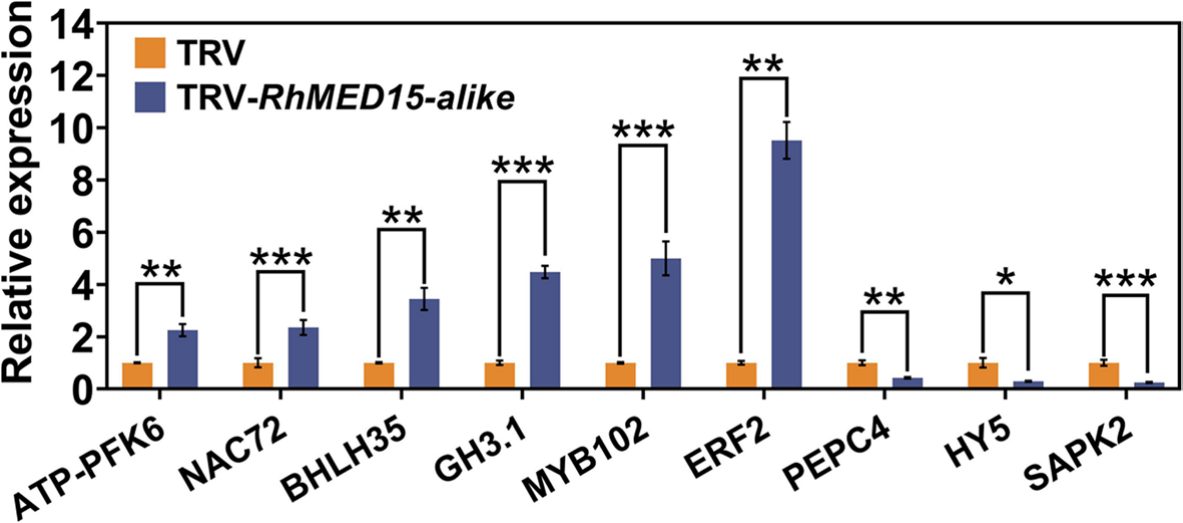
Quantitative RT‒PCR analysis of the transcript levels of DEGs from the RNA-seq analysis. Nine DEGs were randomly chosen, including 6 upregulated DEGs and 3 downregulated DEGs. The transcript levels of the genes in TRV plants were set as 1. Data are the means ± SDs (*n* = 3). Significance of differences determined by independent samples t test are denoted by asterisks (*p < 0.05; **p < 0.01; ***p < 0.001, two-tailed)

For further validation of the RNA-seq data, nine randomly chosen DEGs were subjected to qRT‒PCR. These genes included six upregulated DEGs (*GH3.1*, *bHLH35*, *ATP-PEK6*, *NAC72*, *MYB102* and *ERF2*) and three downregulated DEGs (*SAPK2*, *HY5*, *PEPC4*). The analysis was conducted on *RhMED15a-like*-silenced and TRV control samples (Fig. 8). The results revealed a high resemblance in the expression patterns of DEGs between the qRT‒PCR and RNA-seq analyses, despite minor variations in FC values. This similarity suggests highly consistent results between the two analytical methods, subsequently confirming the trustworthiness and precision of the RNA-Seq analysis.

## Discussion

Drought, a pervasive abiotic stressor, significantly curtails plant productivity and survival in numerous regions worldwide [1, 4, 5]. Many studies have reported that TFs play a notable role in bolstering drought resistance in plants via regulating the expression of drought-related genes [9, 10]. However, there is minimal research on the involvement of transcription cofactors such as Mediator. The plant Mediator is involved in RNA pol II-mediated transcription [52]. Mediator has crucial functions in various growth, development, stress response and cellular movement processes in plants [19, 26, 53, 54]. To date, the majority of studies on plant Mediator subunits have been conducted with *Arabidopsis*, with minimal research extending to other plant species, such as rice and tomato [55, 56]. In our study, we discovered a new Mediator Tail module subunit, *RhMED15a-like*, in rose and investigated how it influences the plant response to water stress in *Arabidopsis* and rose.

Multiple Mediator subunits, namely, MED2, MED7, MED8, MED14, MED16, MED17, MED19a, MED25, MED36a, and CDK8, play a role in the response to various abiotic stresses [11, 19]. With respect to water scarcity, *med25* mutants of *Arabidopsis* exhibited drought resistance [33–35], while *Med19a* mutants were less resistant to drought stress, as confirmed by increased water loss rates and reduced survival rates [57]. Compared with the control, the *Arabidopsis cdk8* mutant displayed diminished ABA sensitivity, flawed stomatal apertures, and increased drought stress susceptibility [39]. Our research revealed that *RhMED15a-like* also plays a pivotal role in plant resistance to water stress. *RhMED15a-like* overexpression heightened osmotic stress tolerance in transgenic *Arabidopsis*, as indicated by an increased germination rate, extended root length, and increased fresh weight under mannitol treatment (Fig. 4). In contrast, *RhMED15a-like* silencing decreased tolerance to drought stress in rose, as verified by an escalated leaf wilting rate, increased MDA content, and reduced survival rate (Fig. 5). These findings suggest that *RhMED15a-like* functions as a positive regulator of plant tolerance to water stress. These results not only deepen our knowledge of the functions of Mediator subunits in plants but also shed light on the molecular mechanisms underpinning tolerance to drought stress in rose.

In addition, a subset of mediator subunits, such as MED15, has been previously reported to participate in development processes. Notably, *med15*/*nrb4-4 Arabidopsis* plants exhibited a reduced size and a lower flower count but, conversely, an increase in additional stems post-bolting compared to the wild type [58]. In contrast, no significant differences were observed in plant height between *RhMED15a-like-*silenced rose plants and TRV controls or between the sizes of *RhMED15a-like*-overexpressing *Arabidopsis* and VC plants (data not shown). The phenotypic disparities between *RhMED15a-like*-silenced rose and *med15 Arabidopsis* potentially stemmed from the divergent roles that MED15s might play in various plant species and at different plant developmental stages, as well as from the efficiency of gene silencing. Therefore, an in-depth investigation is required to determine whether *RhMED15a-like* contributes to the growth and development of rose plants.

Previous reports have shown that drought-related gene expression, which can influence drought resistance, can be regulated in genetically modified plants or mutants during water stress [34, 59]. For example, the expression levels of *RD29A*, *RD29B*, and *DREB2A* notably increase under drought treatment in *med25 Arabidopsis*, corroborating its drought-resistant phenotype [34]. CDK8, another Mediator subunit, serves as a positive regulator of both drought response pathways and ABA signalling in *Arabidopsis* [39]. ABA-responsive genes, including *DREB2A*, *RD29B*, *COR27*, and *RAP2.6*, were less strongly induced in the *cdk8* mutant than in the wild type. Our study revealed significantly lower transcript levels of drought-responsive genes, including stress/ABA-responsive genes (*RD29A*, *RhP5CS*, *ERD14*) [60–62], an ABA synthesis gene (*RhNCED1*) [63], and an ABA-independent gene (*RhDREB1B*) [64], in *RhMED15a-like*-silenced rose than in TRV controls during drought condition, which aligned with the drought-sensitive phenotype of *RhMED15a-like*-silenced rose plants (Figs. 5 and 6). Therefore, *RhMED15a-like* may be involved in rose drought tolerance through the modulation of gene expression in ABA-dependent as well as ABA-independent pathways.

Some phytohormones, notably ABA, JA, SA, and ethylene, have been shown to either independently or synergistically manage drought responses in plants [4, 42, 65, 66]. ABA, in particular, is a crucial stress-signalling hormone that increases in concentration during drought stress periods, acting as the primary hormonal signalling agent for plants under water stress conditions [67, 68]. Our current study revealed differential expression of two significant genes associated with ABA signal transduction, *phosphatase 2C* (*PP2C*) and *serine/threonine-protein kinase* (*SAPK*), between *RhMED15a-like*-silenced rose and TRV controls (Table 1). This suggested the possibility of ABA signalling pathway activation due to *RhMED15a-like* silencing. Similarly, rice *sapk2* mutants were found to be more susceptible to drought stress compared to their wild counterparts, indicating that *SAPK2* can increase the tolerance of rice plants [69]. In contrast, overexpression of *Zea mays ZmPP2C* in *Arabidopsis* resulted in decreased salt and drought tolerance [70]. In this context, our RNA-seq analysis revealed a substantial suppression of *SAPK2-like* expression and an induction of *PP2C 77* due to *RhMED15a-like* silencing in rose plants, consistent with the observed reduction in drought resistance in *RhMED15a-like*-silenced plants. With regard to JA, growing evidence suggests that JA primarily regulates plant drought resistance by modulating stomatal closure and transpiration loss [71–73]. Our results showed that within the JA signalling pathway, 3 TIFY genes and 2 MYC genes, which include MYC2, a critical regulator of the JA pathway, were significantly induced in *RhMED15a-like*-silenced rose (Table 1). It was previously reported that the levels of JA-Ile increased in petals under dehydration, weakening the ability for osmotic adjustment in petal cells and thereby reducing the tolerance to dehydration stress in rose [42, 66]. Consequently, the induction of these genes by the silencing of *RhMED15a-like* was consistent with the drought-sensitive phenotype of the silenced plants. Nevertheless, further evidence is necessary to explore whether *RhMED15a-like* plays a role in regulating JA and/or ABA signalling under drought conditions in rose.

While *RhMED15a-like* is instrumental in enhancing drought stress tolerance in rose, its transcript levels were significantly downregulated following dehydration stress and stress-related hormone treatments (Fig. 2). Parallel outcomes have been documented in related studies [74–76]. For instance, *Arabidopsis PUB22* and *PUB23*, two analogous E3 ubiquitin ligases, are swiftly and simultaneously triggered by drought stress. Intriguingly, *pub22* and *pub23* mutants, which are functionally deficient, exhibited markedly improved drought tolerance. Subsequent research revealed that the protein products of these two genes collaboratively regulate the drought signalling pathway through the ubiquitination of RPN12a, a component of the 19S regulatory particle in the 26S proteasome [74]. Similarly, the transcriptional activity of *osmotically responsive gene 4* (*LOS4*) remained unaffected by cold stress, while its mutant demonstrated increased sensitivity to such stress. Further experimental evidence showed that the regulation of cold-responsive genes and cold sensitivity in *los4* plants were triggered by severe defects in mRNA export [77]. These reports imply that the role of genes in environmental stress resistance is not consistent with their expression profiles under stressful conditions due to the multifaceted molecular mechanisms interwoven with regulation processes during environmental stress responses [78, 79]. Given the functional attributes of the Mediator Tail subunit, RhMED15a-like is likely to be instrumental in conferring drought resistance. It may achieve this through interacting with hormonal signalling elements and a multitude of TFs, thereby altering the expression of numerous genes responsive to drought and enhancing rose tolerance to such stress. For instance, MED25, a Mediator subunit in the Tail module, contrarily regulates drought resistance through interactions with drought stress-induced proteins such as DREB2A, PHOSPHATE STARVATION RESPONSE LIKE1 (PHL1), and ZINC FINGER HOMEODOMAIN 1 (ZFHD1) [34, 80]. Similarly, MED15a homologues have been found to interact with lots of TFs [47]. In *Arabidopsis*, interactome analysis revealed forty-five proteins that interact with the KIX domain of AtMed15a [47]. Our study also revealed a KIX domain in the deduced protein of RhMed15a-like (Fig. S2). Moreover, subcellular localization analysis revealed that RhMED15a-like is present in the nucleus and plasma membrane (Fig. 3), which is similar to the localization pattern of AtMed15a and OsMED15a [81]. Both AtMed15a and OsMED15a are known to interact with nuclear and nonnuclear proteins localized at various cellular sites, indicating that these Mediator subunits may perform additional roles beyond their involvement in the Mediator complex. Our aim in future investigations will be to screen the interactors of RhMED15a-like which play an important role in the response to drought stress in rose.

## Conclusions

In conclusion, the novel Mediator subunit *RhMED15a-like* was successfully isolated and characterized from rose plants. Upon exposure to dehydration stress and treatment with both ABA and MeJA, the transcript level of *RhMED15a-like* was markedly downregulated. Overexpression of *RhMED15a-like* in *Arabidopsis* resulted in enhanced osmotic stress tolerance, whereas silencing of *RhMED15a-like* in rose plants diminished drought stress tolerance. The expression of several stress-responsive genes was lower in the TRV-*RhMED15a-like* plants than in the TRV plants under drought stress. Furthermore, many DEGs involved in the JA and ABA signalling pathways were induced in these silenced plants. Our findings suggest that *RhMED15a-like* ameliorates tolerance to drought stress in rose, possibly through modulating the expression of drought-responsive genes in concert with genes associated with stress-related hormone signalling pathways. In future applications, *RhMED15a-like* has potential as a candidate gene for manipulation via genetic engineering techniques to bolster the drought tolerance of rose.

### Supplementary Information

**Additional file 1: Fig. S1.** Expression of *RhMED15a-like* in the transcriptome of rose petals subjected to dehydration treatment (0 h/24 h). **Fig. S2.** Comparison of the amino acid sequences of RhMED15a-like with MED15s from other plants. **Fig. S3.** Heat map of correlation analysis of expression (a) and principal component analysis (b) between every pair of biological replicates.

**Additional file 2: Table S1.** Primer used in this study. **Table S2.** Summary of RNA-seq data sets. **Table S3.** DEGs enriched in GO term relative to oxidative stress.

## Data Availability

The datasets supporting the conclusions of this article are included within the article and its additional files, and further inquiries can be directed to the corresponding author. The raw sequence reads of RNA-seq were deposited into the NCBI SRA database under accession no. PRJNA963058 (https://www.ncbi.nlm.nih.gov/sra/?term=PRJNA963058).

## References

[1] Chinnusamy V, Zhu JK (2009). Epigenetic regulation of stress responses in plants. Curr Opin Plant Biol.

[2] Xiong L, Zhu JK (2002). Molecular and genetic aspects of plant responses to osmotic stress. Plant Cell Environ.

[3] Zhang H, Zhu J, Gong Z, Zhu JK (2022). Abiotic stress responses in plants. Nat Rev Genet.

[4] Gupta A, Rico-Medina A, Cano-Delgado AI (2020). The physiology of plant responses to drought. Science.

[5] di Pietro M, Vialaret J, Li GW, Hem S, Prado K, Rossignol M (2013). Coordinated post-translational responses of aquaporins to abiotic and nutritional stimuli in *Arabidopsis* roots. Mol Cell Proteomics.

[6] Bailey-Serres J, Parker JE, Ainsworth EA, Oldroyd GED, Schroeder JI (2019). Genetic strategies for improving crop yields. Nature.

[7] Dinneny JR (2019). Developmental responses to water and salinity in root systems. Annu Rev Cell Dev Biol.

[8] Rellán-Álvarez R, Lobet G, Dinneny JR (2016). Environmental control of root system biology. Annu Rev Plant Biol.

[9] Hirayama T, Shinozaki K (2010). Research on plant abiotic stress responses in the post-genome era: past, present and future. Plant J.

[10] Shinozaki K, Yamaguchi-Shinozaki K (2007). Gene networks involved in drought stress response and tolerance. J Exp Bot.

[11] Chen J, Yang S, Fan B, Zhu C, Chen Z (2022). The Mediator complex: A central coordinator of plant adaptive responses to environmental stresses. Int J Mol Sci.

[12] Soutourina J (2018). Transcription regulation by the Mediator complex. Nat Rev Mol Cell Biol.

[13] Malik S, Roeder RG (2010). The metazoan Mediator co-activator complex as an integrative hub for transcriptional regulation. Nat Rev Genet.

[14] Flanagan PM, Kelleher RJ, Sayre MH, Tschochner H, Kornberg RD (1991). A mediator required for activation of RNA polymerase II transcription in vitro. Nature.

[15] Kelleher RJ, Flanagan PM, Kornberg RD (1990). A novel mediator between activator proteins and the RNA polymerase II transcription apparatus. Cell.

[16] Bäckström S, Elfving N, Nilsson R, Wingsle G, Björklund S (2007). Purification of a plant mediator from *Arabidopsis thaliana* identifies PFT1 as the Med25 subunit. Mol Cell.

[17] Fondell JD, Ge H, Roeder RG (1996). Ligand induction of a transcriptionally active thyroid hormone receptor coactivator complex. Proc Natl Acad Sci U S A.

[18] Bourbon HM (2008). Comparative genomics supports a deep evolutionary origin for the large, four-module transcriptional mediator complex. Nucleic Acids Res.

[19] Crawford T, Karamat F, Lehotai N, Rentoft M, Blomberg J, Strand A (2020). Specific functions for Mediator complex subunits from different modules in the transcriptional response of *Arabidopsis thaliana* to abiotic stress. Sci Rep.

[20] Nagulapalli M, Maji S, Dwivedi N, Dahiya P, Thakur JK (2016). Evolution of disorder in Mediator complex and its functional relevance. Nucleic Acids Res.

[21] Allen BL, Taatjes DJ (2015). The Mediator complex: a central integrator of transcription. Nat Rev Mol Cell Biol.

[22] Buendía-Monreal M, Gillmor CS (2016). Mediator: A key regulator of plant development. Dev Biol.

[23] Cai G, Imasaki T, Yamada K, Cardelli F, Takagi Y, Asturias FJ (2010). Mediator head module structure and functional interactions. Nat Struct Mol Biol.

[24] Soutourina J, Wydau S, Ambroise Y, Boschiero C, Werner M (2011). Direct interaction of RNA polymerase II and mediator required for transcription in vivo. Science.

[25] Zhu X, Chen L, Carlsten JO, Liu Q, Yang J, Liu B (2015). Mediator tail subunits can form amyloid-like aggregates in vivo and affect stress response in yeast. Nucleic Acids Res.

[26] Malik N, Agarwal P, Tyagi A (2017). Emerging functions of multi-protein complex Mediator with special emphasis on plants. Crit Rev Biochem Mol Biol.

[27] Ito J, Fukaki H, Onoda M, Li L, Li C, Tasaka M (2016). Auxin-dependent compositional change in Mediator in ARF7- and ARF19-mediated transcription. Proc Natl Acad Sci U S A.

[28] Hemsley PA, Hurst CH, Kaliyadasa E, Lamb R, Knight MR, De Cothi EA (2014). The Arabidopsis mediator complex subunits MED16, MED14, and MED2 regulate mediator and RNA polymerase II recruitment to CBF-responsive cold-regulated genes. Plant Cell.

[29] Ohama N, Moo TL, Chua NH (2021). Differential requirement of MED14/17 recruitment for activation of heat inducible genes. New Phytol.

[30] Knight H, Mugford SG, Ulker B, Gao D, Thorlby G, Knight MR (2009). Identification of SFR6, a key component in cold acclimation acting post-translationally on CBF function. Plant J.

[31] Blomberg J, Aguilar X, Brännström K, Rautio L, Olofsson A, Wittung-Stafshede P (2012). Interactions between DNA, transcriptional regulator Dreb2a and the Med25 mediator subunit from *Arabidopsis thaliana* involve conformational changes. Nucleic Acids Res.

[32] Chen R, Jiang H, Li L, Zhai Q, Qi L, Zhou W (2012). The Arabidopsis mediator subunit MED25 differentially regulates jasmonate and abscisic acid signaling through interacting with the MYC2 and ABI5 transcription factors. Plant Cell.

[33] An C, Li L, Zhai Q, You Y, Deng L, Wu F (2017). Mediator subunit MED25 links the jasmonate receptor to transcriptionally active chromatin. Proc Natl Acad Sci U S A.

[34] Elfving N, Davoine C, Benlloch R, Blomberg J, Brannstrom K, Muller D (2011). The *Arabidopsis thaliana* Med25 mediator subunit integrates environmental cues to control plant development. Proc Natl Acad Sci U S A.

[35] Liu Y, Du M, Deng L, Shen J, Fang M, Chen Q (2019). MYC2 Regulates the termination of jasmonate signaling via an autoregulatory negative feedback loop. Plant Cell.

[36] Lai Z, Schluttenhofer CM, Bhide K, Shreve J, Thimmapuram J, Lee SY (2014). MED18 interaction with distinct transcription factors regulates multiple plant functions. Nat Commun.

[37] Li T, Wu XY, Li H, Song JH, Liu JY (2016). A dual-function transcription factor, AtYY1, is a novel negative regulator of the Arabidopsis ABA response network. Mol Plant.

[38] Zhu Y, Wang B, Tang K, Hsu CC, Xie S, Du H (2017). An Arabidopsis Nucleoporin NUP85 modulates plant responses to ABA and salt stress. PLoS Genet.

[39] Zhu Y, Huang P, Guo P, Chong L, Yu G, Sun X (2020). CDK8 is associated with RAP2.6 and SnRK2.6 and positively modulates abscisic acid signaling and drought response in Arabidopsis. New Phytol.

[40] Bendahmane M, Dubois A, Raymond O, Bris ML (2013). Genetics and genomics of flower initiation and development in roses. J Exp Bot.

[41] Li W, Fu L, Geng Z, Zhao X, Liu Q, Jiang X (2021). Physiological characteristic changes and full-length transcriptome of Rose (*Rosa chinensis*) roots and leaves in response to drought stress. Plant Cell Physiol.

[42] Zhang S, Feng M, Chen W, Zhou X, Lu J, Wang Y (2019). In rose, transcription factor PTM balances growth and drought survival via PIP2;1 aquaporin. Nat Plants.

[43] Clough SJ, Bent AF (1998). Floral dip: a simplified method for Agrobacterium-mediated transformation of *Arabidopsis thaliana*. Plant J.

[44] Chen W, Yin X, Wang L, Tian J, Yang R, Liu D (2013). Involvement of rose aquaporin RhPIP1;1 in ethylene-regulated petal expansion through interaction with RhPIP2;1. Plant Mol Biol.

[45] Paysan-Lafosse T, Blum M, Chuguransky S, Grego T, Pinto BL, Salazar GA (2023). InterPro in 2022. Nucleic Acids Res.

[46] Krogh A, Larsson B, von Heijne G, Sonnhammer EL (2001). Predicting transmembrane protein topology with a hidden Markov model: application to complete genomes. J Mol Biol.

[47] Kumar V, Waseem M, Dwivedi N, Maji S, Kumar A, Thakur JK (2018). KIX domain of AtMed15a, a Mediator subunit of Arabidopsis, is required for its interaction with different proteins. Plant Signal Behav.

[48] Danquah A, de Zelicourt A, Colcombet J, Hirt H (2014). The role of ABA and MAPK signaling pathways in plant abiotic stress responses. Biotechnol Adv.

[49] Nadarajah K, Abdul Hamid NW, Abdul Rahman NSN (2021). SA-mediated regulation and control of abiotic stress tolerance in rice. Int J Mol Sci.

[50] Wang J, Song L, Gong X, Xu J, Li M (2020). Functions of jasmonic acid in plant regulation and response to abiotic stress. Int J Mol Sci.

[51] Nelson BK, Cai X, Nebenfuhr A (2007). A multicolored set of in vivo organelle markers for co-localization studies in Arabidopsis and other plants. Plant J.

[52] Conaway RC, Conaway JW (2011). Function and regulation of the Mediator complex. Curr Opin Genet Dev.

[53] Samanta S, Thakur JK (2015). Importance of Mediator complex in the regulation and integration of diverse signaling pathways in plants. Front Plant Sci.

[54] Chong L, Guo P, Zhu Y (2020). Mediator complex: a pivotal regulator of ABA signaling pathway and abiotic stress response in plants. Int J Mol Sci.

[55] Wang Y, Liang H, Chen G, Liao C, Wang Y, Hu Z (2019). Molecular and phylogenetic analyses of the mediator subunit genes in *Solanum lycopersicum*. Front Genet.

[56] Zhang H, Zheng D, Yin L, Song F, Jiang M (2021). Functional analysis of *OsMED16* and *OsMED25* in response to biotic and abiotic stresses in rice. Front Plant Sci.

[57] Li X, Yang R, Gong Y, Chen H (2018). The *Arabidopsis* Mediator complex subunit MED19a is involved in ABI5-mediated ABA responses. J Plant Biol.

[58] Canet JV, Dobón A, Tornero P (2012). Non-recognition-of-BTH4, an *Arabidopsis* mediator subunit homolog, is necessary for development and response to salicylic acid. Plant Cell.

[59] Ju YL, Yue XF, Min Z, Wang XH, Fang YL, Zhang JX (2020). VvNAC17, a novel stress-responsive grapevine (Vitis vinifera L.) NAC transcription factor, increases sensitivity to abscisic acid and enhances salinity, freezing, and drought tolerance in transgenic Arabidopsis. Plant Physiol Biochem.

[60] Kiyosue T, Yamaguchi-Shinozaki K, Shinozaki K (1994). Characterization of two cDNAs (*ERD10* and *ERD14*) corresponding to genes that respond rapidly to dehydration stress in *Arabidopsis thaliana*. Plant Cell Physiol.

[61] Msanne J, Lin J, Stone JM, Awada T (2011). Characterization of abiotic stress-responsive *Arabidopsis thaliana RD29A* and *RD29B* genes and evaluation of transgenes. Planta.

[62] Strizhov N, Abrahám E, Okrész L, Blickling S, Zilberstein A, Schell J (1997). Differential expression of two P5CS genes controlling proline accumulation during salt-stress requires ABA and is regulated by ABA1, ABI1 and AXR2 in *Arabidopsis*. Plant J.

[63] Lee SU, Mun BG, Bae EK, Kim JY, Kim HH, Shahid M (2021). Drought stress-mediated transcriptome profile reveals NCED as a key player modulating drought tolerance in *Populus davidiana*. Front Plant Sci.

[64] Wei T, Deng K, Gao Y, Liu Y, Yang M, Zhang L (2016). *Arabidopsis DREB1B* in transgenic *Salvia miltiorrhiza* increased tolerance to drought stress without stunting growth. Plant Physiol Biochem.

[65] Llanes A, Andrade A, Alemano S, Luna M (2016). Alterations of endogenous hormonal levels in plants under drought and salinity. Am J Plant Sci.

[66] Fan Y, Liu J, Zou J, Zhang X, Jiang L, Liu K (2020). The RhHB1/RhLOX4 module affects the dehydration tolerance of rose flowers (*Rosa hybrida*) by fine-tuning jasmonic acid levels. Hortic Res.

[67] Chen K, Li GJ, Bressan RA, Song CP, Zhu JK, Zhao Y (2020). Abscisic acid dynamics, signaling, and functions in plants. J Integr Plant Biol.

[68] Seki M, Umezawa T, Urano K, Shinozaki K (2007). Regulatory metabolic networks in drought stress responses. Curr Opin Plant Biol.

[69] Lou D, Wang H, Liang G, Yu D (2017). OsSAPK2 confers abscisic acid sensitivity and tolerance to drought stress in rice. Front Plant Sci.

[70] Liu L, Hu X, Song J, Zong X, Li D, Li D (2009). Over-expression of a Zea mays L. protein phosphatase 2C gene (ZmPP2C) in Arabidopsis thaliana decreases tolerance to salt and drought. J Plant Physiol.

[71] Daszkowska-Golec A, Szarejko I (2013). Open or close the gate - stomata action under the control of phytohormones in drought stress conditions. Front Plant Sci.

[72] Mahouachi J, Arbona V, Gómez-Cadenas A (2007). Hormonal changes in papaya seedlings subjected to progressive water stress and re-watering. Plant Growth Regul.

[73] Pedranzani H, Sierra-de-Grado R, Vigliocco A, Miersch O, Abdala G (2007). Cold and water stresses produce changes in endogenous jasmonates in two populations of *Pinus pinaster* Ait. Plant Growth Regul.

[74] Cho SK, Ryu MY, Song C, Kwak JM, Kim WT (2008). *Arabidopsis* PUB22 and PUB23 are homologous U-Box E3 ubiquitin ligases that play combinatory roles in response to drought stress. Plant Cell.

[75] Ciesla A, Mitula F, Misztal L, Fedorowicz-Stronska O, Janicka S, Tajdel-Zielinska M (2016). A role for barley calcium-dependent protein kinase CPK2a in the response to drought. Front Plant Sci.

[76] Wang N, Liu Y, Cai Y, Tang J, Li Y, Gai J (2020). The soybean U-box gene GmPUB6 regulates drought tolerance in *Arabidopsis thaliana*. Plant Physiol Biochem.

[77] Gong Z, Dong CH, Lee H, Zhu J, Xiong L, Gong D (2005). A DEAD box RNA helicase is essential for mRNA export and important for development and stress responses in *Arabidopsis*. Plant Cell.

[78] Floris M, Mahgoub H, Lanet E, Robaglia C, Menand B (2009). Post-transcriptional regulation of gene expression in plants during abiotic stress. Int J Mol Sci.

[79] Guo J, Wang S, Valerius O, Hall H, Zeng Q, Li JF (2011). Involvement of *Arabidopsis* RACK1 in protein translation and its regulation by abscisic acid. Plant Physiol.

[80] Zhai Q, Deng L, Li C (2020). Mediator subunit MED25: at the nexus of jasmonate signaling. Curr Opin Plant Biol.

[81] Dwivedi N, Maji S, Waseem M, Thakur P, Kumar V, Parida SK (2019). The Mediator subunit OsMED15a is a transcriptional co-regulator of seed size/weight-modulating genes in rice. Biochim Biophys Acta Gene Regul Mech.

