# Supplementary material for: RhMED15a-like, a subunit of the Mediator complex, is involved in the drought stress response in Rosa hybrida
Source: BMC Plant Biol. 2024 Apr 30;24:351. doi: 10.1186/s12870-024-05059-8 (PMC11059607; doi:10.1186/s12870-024-05059-8)
Supplement: Supplementary file 1 — Additional file 1: Fig. S1. Expression of RhMED15a-like in the transcriptome of rose petals subjected to dehydration treatment (0 h/24 h). Fig. S2. Comparison of the amino acid sequences of RhMED15a-like with MED15s from other plants. Fig. S3. Heat map of correlation analysis of expression (a) and principal component analysis (b) between every pair of biological replicates. [file 12870_2024_5059_MOESM1_ESM.docx]

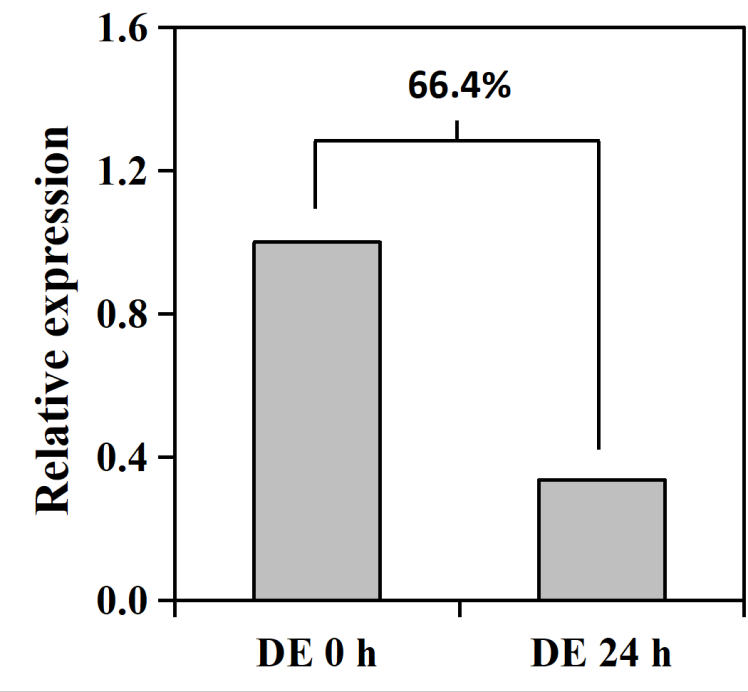
**Fig. S1** Expression of *RhMED15a-like* in the transcriptome of rose petals subjected to dehydration treatment (0 h/24 h).


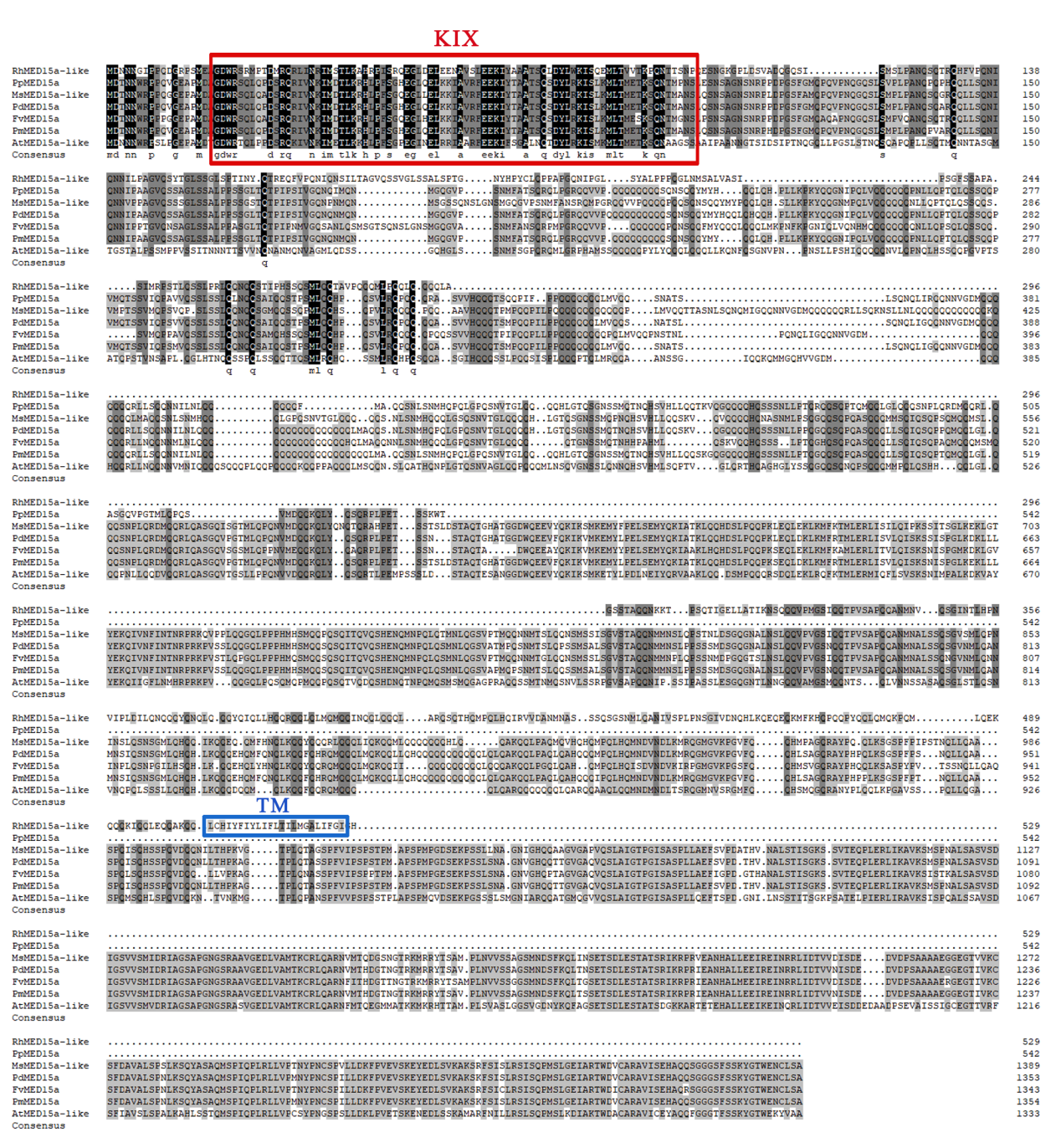


**Fig. S2** Comparison of the amino acid sequences of RhMED15a-like with MED15s from other plants. Identical residues are shown on a black background, and conservative substitutions are shown on a gray background. The conserved KIX domain and transmembrane domain are indicated by red and blue box, respectively. KIX, KIX domain; TM, transmembrane domain. RhMED15a-like (*Rosa hybrida*; XP_024157462.1), PpMED15a (*Prunus persica*; XP_020422007.1), MsMED15a-like (*Malus sylvestris*, XP_050110418.1), PdMED15a (*Prunus dulcis*; XP_034219549.1), FvMED15a (*Fragaria vesca subsp*; XP_011461616.1), PmMED15a (*Prunus mume*; XP_008218603.1), and AtMED15a-like (*Arabidopsis thaliana*; NP_173030.1).


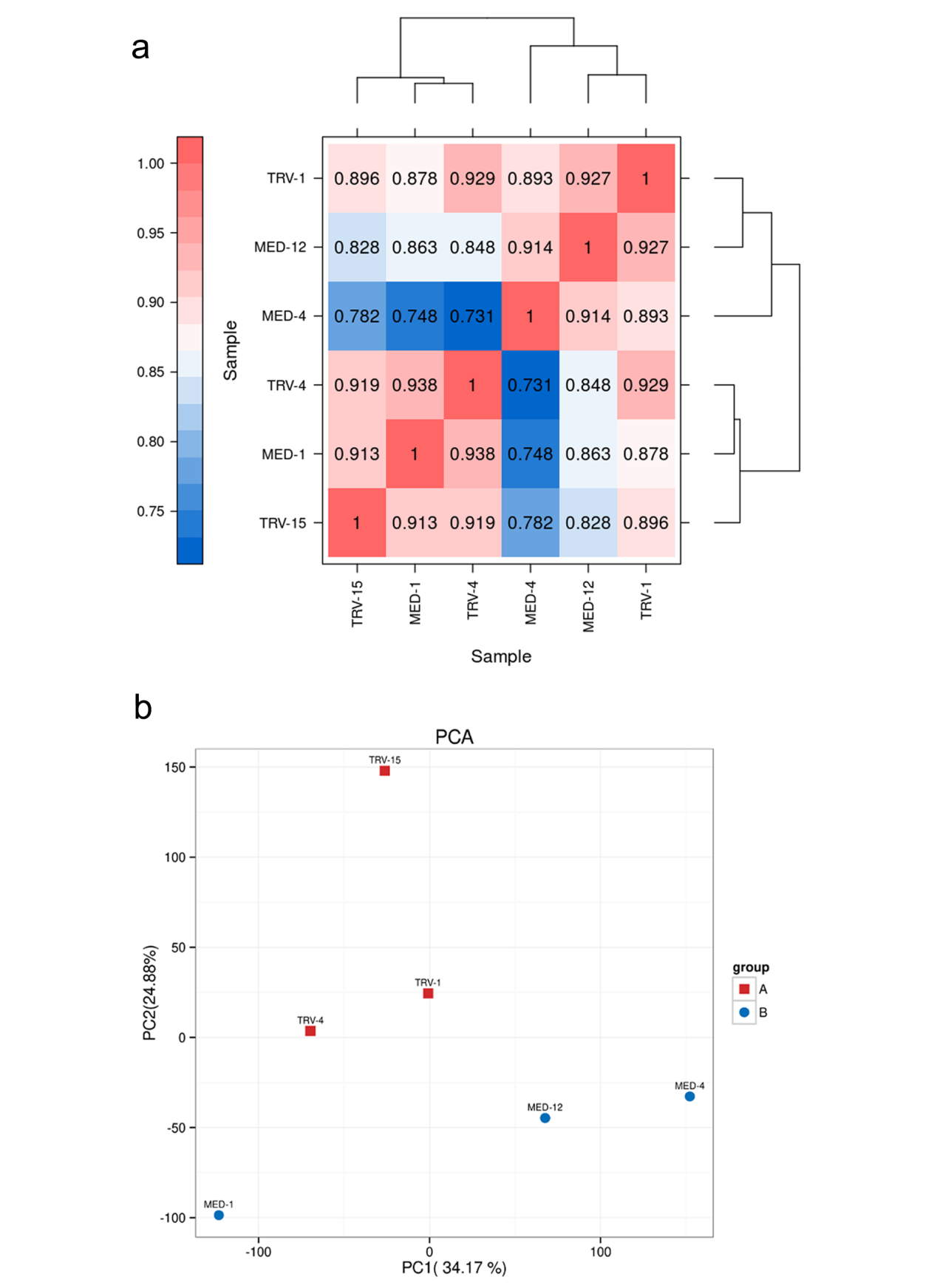


**Fig. S3 Heat map of correlation analysis of expression (a) and principal component analysis (b) between every pair of biological replicates.** TRV, TRV control; MED, TRV-*RhMED15a-like*.
