# Supplementary material for: RhMED15a-like, a subunit of the Mediator complex, is involved in the drought stress response in Rosa hybrida
Source: BMC Plant Biol. 2024 Apr 30;24:351. doi: 10.1186/s12870-024-05059-8 (PMC11059607; doi:10.1186/s12870-024-05059-8)
Supplement: Supplementary file 2 — Additional file 2: Table S1. Primer used in this study. Table S2. Summary of RNA-seq data sets. Table S3. DEGs enriched in GO term relative to oxidative stress. [file 12870_2024_5059_MOESM2_ESM.docx]

**Table S1** Primer used in this study

| **Primer name** | **Nucleotide sequence（5′-3′）** |
| --- | --- |
| **For vector construction** | |
| *RhMED15a-like*-F  *RhMED15a-like*-R | CTACAGACGAGAATCGGTGAT  CCAGTTGTCCCTGTGAAAC |
| pCambia 2300s-*RhMED15a-like*-F  pCambia 2300s-*RhMED15a-like*-R | ATGGATAACAACAATGGCATTCC  TCAATGGCCTATCCCGAAG |
| pCambia 2300s-*eGFP*-F  pCambia 2300s-*eGFP*-R | CTCTCGAGCTTTCGCGAGCTCATGGTGAGCAAGGGCGAGG  TCTAGAGGATCCCCGGGTACCCTTGTACAGCTCGTCCATGCC |
| pCambia 2300s-*RhMED15a-like*-*eGFP*-R | attgttgttatccatggtaccCTTGTACAGCTCGTCCATGCC |
| pCambia 2300s-F  pCambia 2300s-R | CCACCCACGAGGAGCA  TGTGGAATTGTGAGCGGAT |
| TRV1-F  TRV1-R | TTACAGGTTATTTGGGCTAG  CCGGGTTCAATTCCTTATC |
| TRV2-F  TRV2-R | TGGGAGATGATACGCTGTT  CCTAAAACTTCAGACACGGAT |
| TRV2-*RhMED15a-like*-F  TRV2-*RhMED15a-like*-R | GTGAGTAAGGTTACCGAATTCCATACAGAATAACATTCTGCCTGCT  CGTGAGCTCGGTACCGGATCCGTCGCATAATAGATGCTGGAGCA |
| **For qRT-PCR** | |
| *RhUBI2*-RT-F  *RhUBI2*-RT-R | GCCCTGGTGCGTTCCCAACTG  CCTGCGTGTCTGTCCGCATTG |
| *RhMED15a-like*-RT-F  *RhMED15a-like*-RT-R | ATCGGTGATTTCCAATCGTCTC  TTTGCCGCATATCCGTGG |
| *RhDREB1B*-RT-F  *RhDREB1B*-RT-R | GGGAGGAATCGGAAGTTGTTTC  GCTAAATGGAGTAACTCCACAGTGAC |
| *RhRD29A*-RT-F  *RhRD29A*-RT-R | TGTGTGTAGCGAACTAGCGACTT  TCATGCGTTACGTTTTTCCACT |
| *RhP5CS*-RT-F  *RhP5CS*-RT-R | TCACTTACACCCACAAGGACCTC  CAATACGCTAACTAATGTGAGTCGG |
| *RhNCED1*-RT-F  *RhNCED1*-RT-R | GTCTCCCTAGTTACATTTCCACTCG  GGGATCGGCAGAAACCAACT |
| *RhERD14*-RT-F  *RhERD14*-RT-R | CCATCCCAAGACTGAGGAAGAG  TAAAGACGCCAACACCCCAC |
| *ATP-PFK6-*RT-F  *ATP-PFK6-*RT-R | TCCCAATCTTCCGACTTACCC  CGCATGAACATCCTCAGCCT |
| *NAC72-*RT-F  *NAC72-*RT-R | GAAGTAGTTCCCAATACCCAAGCC  CTCCTCCATTTTCCTTCCCATC |
| *bHLH35-*RT-F  *bHLH35-*RT-R | TGGTGAAACTGTGCGAGGTCT  CTCATGGGGCTTTGTGGGT |
| *GH3.1*-RT-F  *GH3.1-*RT-R | GGTTTCCAAGCATTTTAGCCC  CCGTTGACTTTTCCCCGTC |
| *MYB102-*RT-F  *MYB102*-RT-R | TTGACATATTATGATGGGTCGTCG  TCTGGTTGTTGCTGTTGTAGGAGT |
| *ERF2*-RT-F  *ERF2*-RT-R | TCTTAAGACTTCCAAAACTCACAGC  GCTTTCGGTTTCTATGGACATG |
| *PEPC4*-RT-F  *PEPC4*-RT-R | CGACAGTCCGAGTTTCAGATGTT  GATTAGGGATTTCACGCCATTC |
| *HY5*-RT-F  *HY5*-RT-R | GCTCAACAAGCCCGTGAAAG  TCCAAGTTAGAATTGGCATCCTC |
| *SAPK2*-RT-F  *SAPK2*-RT-R | GAAATGTGGGGAGGAGGAAGT  GCTGCTTCCAATGATATGCCTAC |

**Table S2** Summary of RNA-seq data sets

| **Sample Name** | **Clean Reads** | **Clean bases** | **Mapped Reads/%** | **Q30/%** | **GC Content/%** |
| --- | --- | --- | --- | --- | --- |
| TRV-1 | 24,303,780 | 7.25G | 86.9 | 93.4 | 47.73 |
| TRV-4 | 20,920,784 | 6.26G | 87.2 | 93.1 | 47.69 |
| TRV-15 | 20,125,908 | 6.02G | 87.1 | 93.7 | 48.22 |
| MED-1 | 19,919,955 | 5.96G | 87.0 | 92.9 | 47.58 |
| MED-4 | 21,340,076 | 6.38G | 86.7 | 92.0 | 47.43 |
| MED-12 | 19,275,708 | 5.77G | 87.3 | 93.4 | 47.53 |

TRV: control plants; MED: *RhMED15a-like*-silenced plants.

**Table S3** DEGs enriched in GO term relative to oxidative stress

| **Go terms** | **Gene id** | **Gene description** | **log2FC** | ***p* value** |
| --- | --- | --- | --- | --- |
| response to reactive oxygen species | LOC112191440 | 15.7 kDa heat shock protein, peroxisomal | 1.1 | 0.0004 |
|  | LOC112171224 | uncharacterized protein LOC112171224 | 1.4 | 0.0006 |
|  | LOC112169651 | 17.9 kDa class II heat shock protein-like | 1.9 | 0.0056 |
| hydroquinone:oxygen oxidoreductase activity | NewGene_620 | putative laccase | 1.4 | 0.0011 |
|  | LOC112201336 | laccase-17-like | 1.1 | 0.0053 |
|  | LOC112173364 | laccase-11-like | 1.0 | 0.0012 |
|  | LOC112190639 | putative laccase-9 | 1.3 | 0.0054 |
|  | LOC112173083 | laccase-11-like | 1.4 | 0.0000 |
| peroxisome | LOC112180744 | peroxisome biogenesis protein 19-2-like | -1.3 | 0.0034 |
|  | LOC112191440 | 15.7 kDa heat shock protein, peroxisomal | 1.1 | 0.0004 |
|  | LOC112186191 | acyl-coenzyme A oxidase 2, peroxisomal | 1.0 | 0.0056 |
